# The Value of Androgen Measures for Diagnosing Polycystic Ovary Syndrome (PCOS) in an Unselected Population

**DOI:** 10.1007/s43032-024-01702-9

**Published:** 2024-10-17

**Authors:** L. Pace, N. Kummer, M. Wallace, R. Azziz

**Affiliations:** 1 Dept. of Ob/GynHeersink School of Medicine, University of Alabama at Birmingham, Birmingham, AL USA; 2 Heersink School of Medicine, University of Alabama at Birmingham, Birmingham, AL USA; 3 Dept. of Medicine, Heersink School of Medicine, University of Alabama at Birmingham, Birmingham, AL USA; 4 Dept. of Healthcare Organization & Policy, School of Public Health, University of Alabama at Birmingham, Birmingham, AL USA; 5 Dept. of Health Policy, Management, and Behavior, School of Public Health, University at Albany, SUNY, Rensselaer, NY USA

**Keywords:** Androgens, PCOS, Polycystic ovary syndrome, Oligomenorrhea, Diagnosis, Epidemiology, Androgen excess, Hyperandrogenism

## Abstract

**Objective:**

Polycystic Ovary Syndrome (PCOS) is diagnosed by a combination of three features: hyperandrogenism (biochemical and/or clinical), ovulatory dysfunction, and polycystic ovarian morphology, usually detected by ultrasonography. Our study aimed to determine the need for androgen measurements by using hirsutism to establish hyperandrogenism for diagnosing PCOS in a medically unbiased population.

**Materials and Methods:**

We utilized a pre-existing cohort of unselected (medically unbiased) females aged 18–45 years. All underwent a history and physical, including a modified Ferriman-Gallwey (mFG) hirsutism score. Subjects were categorized clinically as eumenorrheic non-hirsute (CONTROLS), menstrual dysfunction only (OLIGO-ONLY), hirsutism only (HIRSUTE-ONLY), or menstrual dysfunction and hirsutism (OLIGO + HIRSUTE). All subjects underwent measurements of androgens using high-quality assays. CONTROLS established the upper normal limit for androgen levels. We defined PCOS using the NIH 1990 criteria.

**Results:**

Of 462 individuals with complete evaluations, 311 (67.3%) were CONTROLS, 71 (15.4%) were OLIGO-ONLY, 64 (13.9%) were HIRSUTE-ONLY, and 16 (3.5%) were OLIGO + HIRSUTE. Neither HIRSUTE-ONLY nor OLIGO-HIRSUTE women required androgen measures to demonstrate hyperandrogenism. Among OLIGO-ONLY, 19 (26.8%) demonstrated hyperandrogenemia without hirsutism, with White women significantly more likely than Black women to demonstrate this.

**Conclusions:**

In our study of medically unbiased reproductive-aged women using the NIH 1990 criteria for PCOS, only 15.4% of women evaluated (those with menstrual dysfunction only) required androgen measurements. In these women only one-quarter demonstrated hyperandrogenemia. These data provide a strategy to minimize the need for androgen assays, including firstly categorizing subjects by clinical presentation and then assessing circulating androgens in the subgroup with menstrual dysfunction only.

## Introduction

Polycystic Ovary Syndrome (PCOS) is a common complex condition which affects 12–14% of women and, while life-long, is most clinically evident in reproductive-aged individuals [1, 2]. Multiple sets of diagnostic criteria have been proposed for PCOS, each of which includes some combination of three clinical features: hyperandrogenism (biochemical and/or clinical), ovulatory dysfunction, which is generally reflected by menstrual dysfunction, and polycystic ovarian morphology, usually detected by ultrasonography [3–8]. Clinical hyperandrogenism has been historically determined using the modified Ferriman-Gallwey (mFG) visual scale for hirsutism alongside evidence of acne and androgenic alopecia [9, 10]. The presence of hirsutism has been described as a strong, reliable indicator of androgen excess, with an estimated 65–75% prevalence in PCOS patients, and significant correlations between androgen levels and mFG score [11–13]. Alternatively, biochemical hyperandrogenism (i.e., hyperandrogenemia) alone correlates poorly with the severity of PCOS symptoms due to these hormones’ cyclic nature, assay quality dependence, and limited test sensitivity and specificity [14]. In addition to the time and effort required to gather data in the diagnostic phase, the estimated cost of initial laboratory evaluation may, in many cases, present a burden to patients and healthcare systems.

We and others have reported on the economic burden of PCOS based on epidemiologic and prevalence data, however, our understanding of the impact of PCOS globally has been limited by the availability of high-quality studies[15]. This is particularly problematic in under-resourced settings, where the limited quality and availability of androgen assays may pose a challenge to diagnosis of hyperandrogenemia [16]. However, it is critical to understand that not all women suspected of PCOS require androgen measurements for diagnosis, particularly those individuals who have clinical evidence of hyperandrogenism, i.e., hirsutism. This concept is reflected in the newly proposed diagnostic algorithm highlighted in the 2023 International Evidence-Based Guidelines for the Assessement and Management of Polycystic Ovarian Syndrome [8], which specify that the assessment of biochemical hyperandrogenism is of greatest value in patients with minimal or no evidence of clinical hyperandrogenism.

In light of the potential burden of androgen assay testing in the diagnosis of PCOS, and in alignment with the newly proposed diagnostic algorithm of the 2023 International Guidelines, we aimed to analyze the frequency with which androgen measurements were necessary for diagnosing PCOS in an unselected patient population [8]. We sought to conduct this analysis using a pre-existing cohort of medically unbiased reproductive-aged women representative of the larger surrounding population in Birmingham, AL where the study was conducted.

## Materials and Methods

### Subjects

This study utilized a pre-existing database containing a cohort of medically unbiased reproductive-aged women representative of the surrounding population in Birmingham, Alabama [1, 2]. Subjects were individuals undergoing a mandatory pre-employment health assessment at the University of Alabama at Birmingham (UAB) between 1998 and 2001 who consented to participation. All consecutive premenopausal females aged 18–45 years were asked to participate and the initial study and data collection were approved by UAB’s Institutional Review Board. Data on this population has been previously reported[1, 2, 17].

Exclusion criteria for the study included those who did not consent to blood draws or did not complete the blood draw and therefore have no serum androgens recorded, those who had undergone menopause, those with a history of prior hysterectomy or bilateral salpingo-oophorectomy, and those found to be pregnant at the time of evaluation. It is worth noting that the use of hormonal contraception or other hormonal therapy, the use of glucocorticoids, the presence of pre-existing thyroid conditions undergoing treatment, and the presence of diabetes and use of insulin sensitizing therapy were not criteria for exclusion from the study.

In our analysis, PCOS diagnosis was defined by NIH 1990 Criteria, or Rotterdam Criteria phenotypes A and B, as we specifically sought to evaluate those subjects who displayed “Classic PCOS” phenotype with evidence of androgen excess. Thus, diagnosis was made if subjects met the criteria of oligo-ovulation, as demonstrated by menstrual dysfunction, and clinical or biochemical hyperandrogenism.

## Study Protocol

All included subjects underwent a complete history and physical examination with a research nurse. Subject history obtained included an extensive menstrual and gynecologic history and prior subjective experience of hirsutism and acne. Patients using hormonal therapy were subjected to additional questioning regarding their pattern of menses prior to initiation of treatment.

Physical examination included a modified Ferriman-Gallwey (mF-G) evaluation to score the presence of terminal hairs over nine noted body areas. Scores from 0 to 4 were assigned in each body area, and all subjects with a score above three by the study nurse were re-examined by a physician. The presence of acne was also noted on examination. Blood was collected from each subject for subsequent hormonal assay.

For purposes of this study, ovulatory dysfunction was defined as eight or fewer menstrual cycles per year, or menses less than 26 or more than 35 days in length. Of note, this study did not include the use of ultrasound for evaluation of ovarian morphology including antral follicle counts or volumes.

Clinical hyperandrogenism was determined to be present if mF-G score was found to be 4 or greater, as previously reported and consistent with the 2023 International Evidence-based Guidelines for the Assessment and Management of PCOS [8, 18]. Serum testing was considered positive for hyperandrogenemia if total and/or free testosterone (T), androstenedione (A4), and/or dehydroepiandrosterone sulfate (DHEAS) exceeded the 95% of the levels of our healthy control group of 311 non-hirsute, eumenorrheic women. Values for upper normal limits were as follows: TT = 80.0 ng/dl (2.78 nmol/liter), FT = 1.0 ng/dl (0.035 nmol/liter), A4 = 2516 pg/ml (8.78 nmol/liter), and DHEAS = 2425 ng/ml (6.58 μmol/liter).

All subjects with possible PCOS underwent further clinical and laboratory testing to exclude mimicking or similar disorders, including thyroid dysfunction, hyperprolactinemia, and 21-hydroxylase deficient non-classic adrenal hyperplasia (NCAH), by measuring TSH, prolactin (PRL), and 17-hydroxyprogesterone (17-HP), respectively. Subjects who underwent androgen testing but did not complete their evaluation to exclude other conditions were still included for this analysis, as they could be designated as having possible PCOS and their requirement for serum androgen evaluation could be adequately evaluated independent of their completion of other testing.

## Hormonal Assays

Serum samples were analyzed for TT, FT, sex hormone-binding globulin (SHBG), A4, and DHEAS. Selected individuals (as above) also underwent measurement for progesterone (P4), PRL, TSH, and 17-HP. Samples were batched at regular intervals for analysis to minimize the impact of inter-assay variability. TT was measured by in-house radioimmunoassay (RIA) and chromatography, and SHBG estimated using competitive binding, and free T was calculated, as previously described[2]. DHEAS, P4, A4, PRL, TSH, and 17-OHP were measured using commercially available RIA kits at the time of data collection (1998), and the intra- and inter-assay variations were previously reported [2].

## Statistical Analysis

For ease of data analysis, subjects were classified into four groups based on their clinical presentation, as outlined in Table 1. Group 1 (CONTROLS) lacked hirsutism or menstrual dysfunction; group 2 (OLIGO-ONLY) had evidence of menstrual dysfunction but lacked hirsutism on exam; group 3 (HIRSUTE-ONLY) were hirsute subjects without menstrual dysfunction, and group 4 (OLIGO + HIRSUTE) had evidence of both menstrual dysfunction and hirsutism on examination. The 95th percentile of CONTROLS for each androgen measured was used as the upper normal limit for the diagnosis of hyperandrogenemia. Characteristics of each phenotypic subgroup including BMI, age, and androgen levels were evaluated using descriptive statistics. Differences in these baseline characteristics and androgen levels were calculated using analysis of variance (ANOVA), and between-group differences in age and racial characteristics were calculated using chi-squared statistics.

**Table 1:** Subject characteristics by clinical presentation

	**Controls**	**Oligo-only**	**Hirsute-only**	**Oligo + Hirsute**	**P value**
**Race**					
Black (n [%])	153 [49.2]	35 [49.3]	38 [59.4]	6 [37.5]	*χ*^*2*^= 4.48, *p* = .61
White (n [%])	150 [48.2]	35 [49.3]	25 [39.1]	9 [56.3]	
Other (n [%])	8 [2.6]	1 [1.4]	1 [1.5]	1 [6.3]	
**BMI (mean, SD)**	26.8, 6.9	25.8, 8.2	26.7, 6.6	33.2, 9.5	*p* = .004
**Age (mean, SD)**	29.5, 7.0	27.8, 6.3	29.0, 6.8	28.2, 7.5	*p* = .27
**mFG score (mean, SD)**	0.6, 0.9	0.8, 1.1	6.9, 3.0	6.8, 2.8	*p* < .001
**TT (mean, SD)**	49.3, 19.4	53.9, 28.4	55.4, 20.8	59.6, 31.4	*p* = .04
**fT (mean, SD)**	0.35, 0.21	0.48, 0.36	0.42, 0.25	0.58, 0.46	*p* < .001
**DHEAS (mean, SD)**	1063.0, 839.7	1357.2, 688.7	1865.0, 1074.1	1946.9, 897.1	*p* < .001
**A4 (mean, SD)**	1232.5, 639.5	1326.6, 677.5	1400.7, 781.9	1401.9, 1412.2	*p* = .26

BMI = body mass index

TT = serum total testosterone level

fT = serum free testosterone level

DHEAS = serum dehydroepiandrosterone sulfate

A4 = serum androstenedione level

## Results

**Fig. 1:**
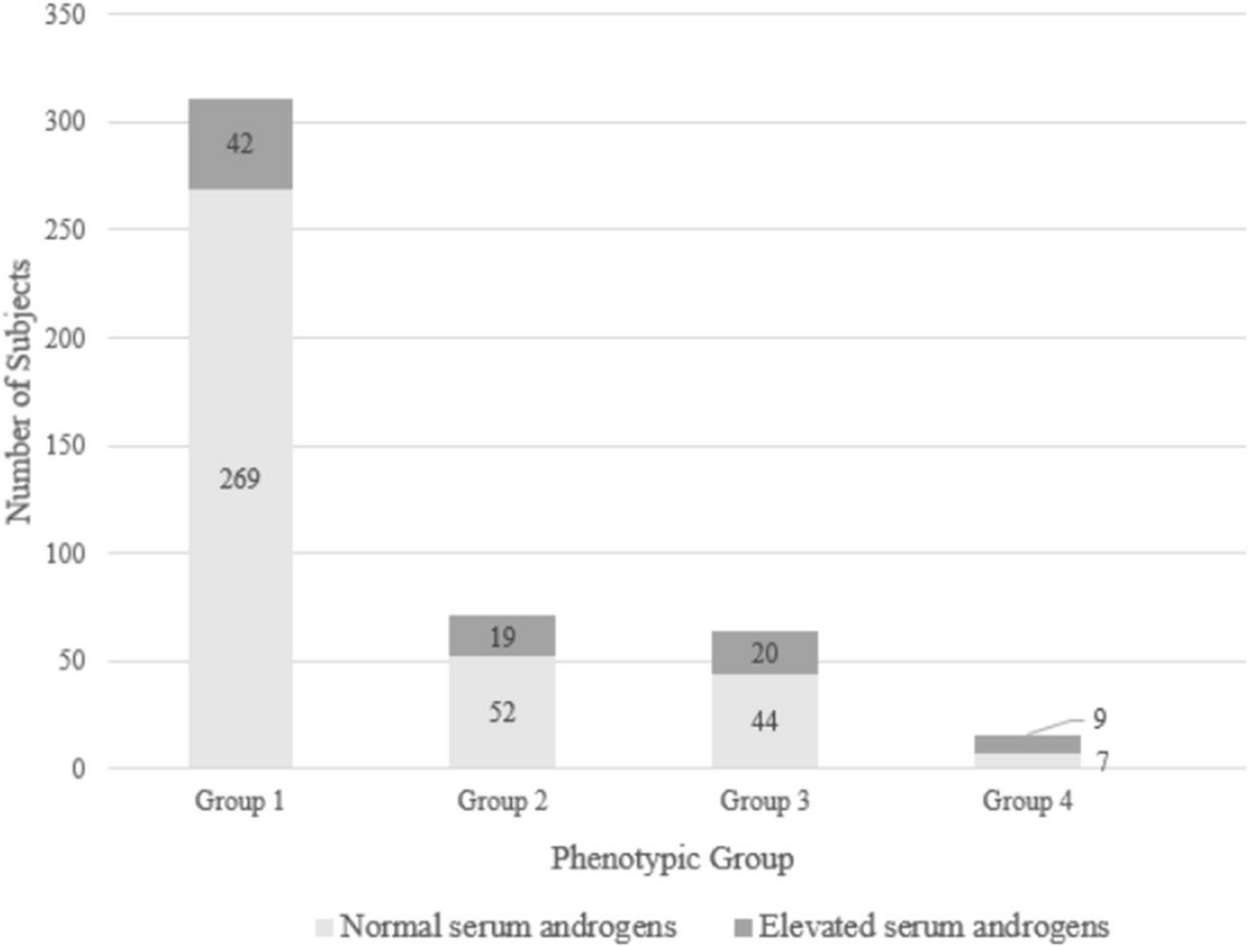
Characterization of subjects using the by clinical presentation groupings outlined in Table 1 facilitates assessing the need for serum androgen testing. For the purposes of this study, serum androgen testing was conducted in all groups, though by definition was only necessary for subjects in group 2 (OLIGO-ONLY)

Of 462 subjects included in this study, a total of 311 (67.3%) were eumenorrheic and non-hirsute (Fig. 1). These subjects were designated as CONTROLS and used to establish upper normal limits for androgen values. Seventy-one (15.4%) of subjects studied demonstrated oligo-ovulation and no hirsutism (OLIGO-ONLY), of which 19 (26.8%) displayed hyperandrogenemia. A total of 64 (13.9%) subjects did not meet criteria for oligo-ovulation based on reported menstrual patterns, but were found to be hirsute (HIRSUTE-ONLY). Finally, sixteen (3.5%) subjects were found to be oligo-ovulatory and hirsute (OLIGO+HIRSUTE). While androgen measurements were not necessary for diagnosis in these latter subgroups, they were obtained for academic and research purposes.

Phenotypic group characteristics showed no statistically significant difference in racial or age distribution among groups, though there were significant differences across groups in average BMI, mFG score, and all androgen measures with the exception of A4 (Table 1). When evaluating the racial differences in hyperandrogenemia, only the OLIGO-ONLY group showed a significant difference, with white race being more strongly associated with androgen elevation. PCOS by NIH 1990 criteria was identified in 19 (26.8%) of women with OLIGO-ONLY who demonstrated hyperandrogenemia and in all 16 (100%) of women with OLIGO+HIRSUTISM. The prevalence of PCOS as defined by the NIH 1990 criteria in this cohort was 7.6% ([19+16]/462).

Racial characteristics of the phenotypic subgroups showed a significantly larger proportion of White subjects in the OLIGO-ONLY group demonstrated hyperandrogenemia (*n* = 14) when compared to Black subjects (*n* = 5) and those of other races (*n* = 1) (*p* = 0.04). The HIRSUTE-ONLY and OLIGO+HIRSUTISM groups did not show a significant difference in racial distribution.

A sub-analysis of subjects by age indicates that of those above age 35 (*n* = 27), 11 required androgen testing, though none of these demonstrated hyperandrogenemia **(**Table 2**)**. Subjects above the age of 35 were not significantly more or less likely to require androgen testing than those aged 18–35 (*p* = 0.53). Rates of hyperandrogenemia were significantly lower among those aged 36–45 than those 18–35 among those with OLIGO-ONLY requiring androgen testing (*n* = 19 vs. 0; *χ*^*2*^ = 4.76, *p* = 0.029) as well as across all phenotypic groups (*n* = 44 vs. 4; *χ*^*2*^ = 4.37, *p* = 0.037).

**Table 2:** Characteristics of non-control subjects by clinical presentation and age

Age	Oligo-only (HA)	Hirsute-only (HA)	Oligo+ Hirsute (HA)	P value
18–35	41 (19)	33 (18)	6 (7)	*χ*^*2*^ = 4.37, *p* = .037
36–45	11 (0)	11 (2)	1 (2)	
	*χ*^*2*^ = 4.76, *p* = .029			

HA = hyperandrogenism

Overall, when using the NIH 1990 criteria to detect PCOS in a medically unbiased population only those women presenting clinically with oligo-ovulation but no hirsutism (i.e., OLIGO-ONLY) would require androgen measures to assess for PCOS. Consequently, only 15.4% (71/462) of the population studied required androgen measures to assess for the presence of biochemical hyperandrogenism.

## Discussion

In this analysis of data from an unselected medically unbiased sample of women, 15.4% were found to require serum androgen testing in their evaluation for PCOS diagnosed by the NIH 1990 criteria (i.e., 'classic PCOS’). This finding supports the 2023 Evidence-based International Guidelines, which now recommend in their diagnostic algorithm for PCOS to test for biochemical hyperandrogenism and exclude other causes only in the absence of clinical hyperandrogenism.

While hirsutism has long been associated with hyperandrogenism, there is also evidence supporting the role of insulin resistance in this condition [19–21]. It is well-established that the presence of insulin resistance is associated with increased rates of other metabolic sequelae for PCOS patients [22]. As such, it is conceivable that its presence in the diagnostic framework of PCOS may portend an even greater quantity of prognostic information than serum androgen values. Moreover, androgen assays capture only a single moment in time due to the physiologic pulsatility of these hormones [23].

Various expert opinions have recommended use of the “highest quality androgen assays” for testing due to the small level of circulating androgens and the similarity in configuration between various sex steroids [6, 15]. The 2023 International Guidelines specifically recommend the use of validated, highly accurate tandem mass spectrometry assays for measuring total testosterone. However, as was acknowledged by Azziz et al. in their 2019 discussion of recommendations for epidemiologic and phenotypic research in PCOS, the cost and availability of quality androgen assays around the globe may, in some cases, be prohibitive for this purpose. The findings of this study provide support to their conclusion that being more selective in the use of androgen assays will not hinder, and is perhaps a preferable method to, detecting PCOS in large-scale epidemiologic studies of this condition.

Another important consideration in the evaluation of PCOS is cost – both in financial and human terms. In 2013, unnecessary services added $210 billion to healthcare spending in the United States [24]. Azziz et al. estimated that in 2004 dollars, the total cost of lab work alone for evaluation of PCOS was $737.54 ($1,366.35 in 2023 dollars) per patient [14]. Beyond this, the intangible costs to patients of delayed diagnosis and emotional uncertainty take a well-documented toll on mental health [25–27]. By phenotypically stratifying patients, as was done in this study, physicians can more expediently communicate to patients their diagnosis of PCOS while also more efficiently and effectively completing their evaluation.

Racial differences in the diagnosis of PCOS remain a complex topic of study given inherent differences in characteristics such as hair growth pattern and BMI as well as systematic healthcare disparities, epigenetic factors, and the complex role of socioeconomic and cultural influences [2, 18, 28]. The finding in this study of higher levels of hyperandrogenemia among oligomenorrheic White women as compared to Black women appears to be novel, however, it remains unclear whether this findings is generalizable to a larger patient population. Research by VanHise et al. has explored the complex interplay of regional, sociodemographic, and racial factors in the expression of PCOS phenotypes [29]. Their study found that hyperandrogenemia was more common among Black women in California than in Alabama, perhaps suggesting that environmental factors play a role in this diagnostic criterion. It is clear that further research is needed to evaluate the individual and combined roles of each of various racial and demographic factors in the development of PCOS as well as its various phenotypic manifestations and sequelae.

Our subgroup analysis by age suggests that the relative utility of biochemical androgen testing is higher in younger subjects who demonstrate oligomenorrhea without hirsutism. While our analysis is limited by a small sample size of women over 35, this finding is supported by prior studies that demonstrate a decrease in hyperandrogenemia associated with PCOS with advancing age [30]. This important limitation should therefore be considered in the evaluation of older women presenting with a complaint of oligomenorrhea without hirsutism, as the utility of serum androgen testing may be lower overall in this population.

Based on the results of this study, it is recommended that high-quality serum androgen assays be reserved for evaluation of PCOS in young women presenting with oligomenorrhea without hirsutism and may be considered in older (> 35 years) women with this presentation. Such an evaluation should be undertaken only after other causes of oligomenorrhea have been excluded by proper evaluation of factors such as TSH, prolactin, and 17-OHP, and possibly FSH and LH, in accordance with the 2023 International Guidelines [8]. Given the direct relationship between increased androgen levels and insulin resistance [31], when taken in consideration with the long-term health sequelae of untreated PCOS such as increased risk for endometrial cancer and cardiovascular disease [32], it is our recommendation that hirsutism be considered an appropriate proxy for serum androgen elevation in the diagnosis and treatment of PCOS. The impact of treatments varying from lifestyle modifications to hormonal and metabolic mediators on insulin resistance and adipose tissue dysfunction in PCOS is well-documented and should be considered early in the treatment of this condition to prevent adverse future health outcomes [33].

Our study benefitted from an unselected, medically non-biased patient population. Socioeconomic bias was also minimized in the study population, as subjects were selected from all staff seeking employment at UAB, which is the single largest employer in the city of Birmingham, and the largest public employer in the state of Alabama. The positions sought by subjects included custodians, secretarial staff, physicians, nursing staff, and a variety of other clerical and support staff. The use of an internal control group of eumenorrheic, non-hirsute women from which the upper-limit of normal for androgen values was established is another strength of this study.

The inclusion of subjects regardless of hormonal contraception, steroid administration, or insulin/glucose sensitization, or thyroid hormone therapy may, admittedly, have impacted results. It was estimated that from 2017–2019 approximately 17.1% of women aged 15–49 in the United States were using oral contraceptive pills, Depo-Provera, vaginal rings, or hormonal contraceptive patches – all of which would impact menstrual history, clinical hirsutism, and serum androgen levels [34]. An additional limitation of this study is the time period over which data was obtained. Since the initial data collection occurred over twenty years ago, it cannot necessarily be concluded to directly reflect current population trends. With the known rise in obesity incidence year over year in the state of Alabama [35], it is certainly conceivable that the incidence of PCOS has also increased. Research efforts to obtain updated data from a similar unselected population are currently ongoing at the original study institution. The study population included is also limited with regard to racial diversity. The vast majority of subjects were White or Black, with other minority races poorly represented. While this is fairly representative of the demographics of Birmingham as recently as the 2020 US Census [36], we recognize that it is not entirely representative of the US population at large.

It is worth noting that the 2023 International Guidelines uphold the Rotterdam 2023 Criteria for diagnosis of PCOS and consider the condition diagnosable in the presence of two out of the following three, after exclusion of other etiologies: 1) oligo-ovulation or anovulation, 2) hyperandrogenism, and 3) polycystic ovarian morphology. We recognize that the definition used in this study is only partially consistent, and far narrower, than that proposed in these guidelines. However, as the new guidelines recommend ultrasound evaluation if patients have only irregular menses or hyperandrogenism, then the categorizations of possible PCOS used in our results remain valid. Nonetheless, we acknowledge the potential utility of a future study analyzing the need for androgen assay in diagnosing PCOS using the 2023 International Guidelines. In this study, the addition of ultrasound would have been helpful for further characterization of subjects in Group 2 (OLIGO-ONLY) and Group 3 (HIRSUTE-ONLY), as those meeting criteria for polycystic ovarian morphology would, excluding other causes, be diagnosed with PCOS.

We conclude that stratification by clinical presentation, particularly the presence of hirsutism, to determine the need for circulating androgen measures is a useful tool in the diagnosis of PCOS. Furthermore, in an unselected patient population, less than one-fifth of patients required serum androgen assay in their evaluation. This finding is directly aligned with the recently published 2023 International Evidence-based Guideline for the Assessment and Management of Polycystic Ovary Syndrome [8]. These evidence-based guidelines state that the presence of hirsutism or clinical hyperandrogenism alone should be considered predictive of biochemical hyperandrogenism and PCOS in adults, alleviating the necessity for assays in all-comers. The findings of this study support a diagnostic algorithm in which serum androgen testing should only be performed in cases where clinical hyperandrogenism is absent, but other markers concerning PCOS are present, such as oligo-ovulation and menstrual dysfunction.

## Data Availability

Raw data were generated at UAB. Derived data supporting the findings of this study are available from the corresponding author RA on request.

## References

[1] Azziz R, Woods KS, Reyna R, Key TJ, Knochenhauer ES, Yildiz BO. The prevalence and features of the polycystic ovary syndrome in an unselected population. J Clin Endocrinol Metab. 2004;89(6):2745–9. 10.1210/jc.2003-032046. 15181052 10.1210/jc.2003-032046

[2] Knochenhauer ES, Key TJ, Kahsar-Miller M, Waggoner W, Boots LR, Azziz R. Prevalence of the polycystic ovary syndrome in unselected black and white women of the southeastern United States: a prospective study. J Clin Endocrinol Metab. 1998;83(9):3078–82. 10.1210/jcem.83.9.5090. 9745406 10.1210/jcem.83.9.5090

[3] Zawadski JK, Dunaif A. Diagnostic criteria for polycystic ovary syndrome: towards a rational approach. In: Dunaif A, Givens JR, Haseltine F, editors. Polycystic ovary syndrome. Boston: Blackwell Scientific; 1992. p. 377–84.

[4] Rotterdam ESHRE/ASRM-Sponsored PCOS Consensus Workshop Group. Revised 2003 consensus on diagnostic criteria and long-term health risks related to polycystic ovary syndrome (PCOS). Hum Reprod. 2004;19(1):41–7. 10.1093/humrep/deh098. 14688154 10.1093/humrep/deh098

[5] Azziz R, et al. Positions statement: criteria for defining polycystic ovary syndrome as a predominantly hyperandrogenic syndrome: an Androgen Excess Society guideline. J Clin Endocrinol Metab. 2006;91(11):4237–45. 10.1210/jc.2006-0178. 16940456 10.1210/jc.2006-0178

[6] Teede HJ, et al. Recommendations from the international evidence-based guideline for the assessment and management of polycystic ovary syndrome. Clin Endocrinol (Oxf). 2018;89(3):251–68. 10.1111/cen.13795. 30024653 10.1111/cen.13795 PMC9052397

[7] Hampton T. NIH panel: Name change, new priorities advised for polycystic ovary syndrome. JAMA. 2013;309(9):863. 10.1001/jama.2013.1236. 23462765 10.1001/jama.2013.1236

[8] Teede HJ, et al. Recommendations From the 2023 International Evidence-based Guideline for the Assessment and Management of Polycystic Ovary Syndrome. J Clin Endocrinol Metab. 2023;108(10):2447–69. 10.1210/clinem/dgad463. 37580314 10.1210/clinem/dgad463 PMC10505534

[9] Yildiz BO, Bolour S, Woods K, Moore A, Azziz R. Visually scoring hirsutism. Hum Reprod Update. 2010;16(1):51–64. 10.1093/humupd/dmp024. 19567450 10.1093/humupd/dmp024 PMC2792145

[10] Grimstad F, Moyer Q, Williams CR, Kremen J. A body-neutral and gender-neutral modified Ferriman-Gallwey diagram. J Pediatr Adolesc Gynecol. 2022;35(3):375–8. 10.1016/j.jpag.2021.10.015. 34748917 10.1016/j.jpag.2021.10.015

[11] Spritzer PM, Marchesan LB, Santos BR, Fighera TM. Hirsutism, normal androgens and diagnosis of PCOS. Diagnostics. 2022;12(8):1922. 36010272 10.3390/diagnostics12081922 PMC9406611

[12] Yang Y, et al. The predictive value of total testosterone alone for clinical hyperandrogenism in polycystic ovary syndrome. Reprod Biomed Online. 2020;41(4):734–42. 10.1016/j.rbmo.2020.07.013. 32912651 10.1016/j.rbmo.2020.07.013

[13] Ruutiainen K, Erkkola R, Kaihola H-L, Santti R, Irjala K. The grade of hirsutism correlated to serum androgen levels and hormonal indices. Acta Obstet Gynecol Scand. 1985;64(8):629–33. 10.3109/00016348509158203. 2938400 10.3109/00016348509158203

[14] Azziz R, Marin C, Hoq L, Badamgarav E, Song P. Health care-related economic burden of the polycystic ovary syndrome during the reproductive life span. J Clin Endocrinol Metab. 2005;90(8):4650–8. 10.1210/jc.2005-0628. 15944216 10.1210/jc.2005-0628

[15] Azziz R, et al. Recommendations for epidemiologic and phenotypic research in polycystic ovary syndrome: an androgen excess and PCOS society resource. Hum Reprod. 2019;34(11):2254–65. 10.1093/humrep/dez185. 31751476 10.1093/humrep/dez185

[16] Rosner W, Auchus RJ, Azziz R, Sluss PM, Raff H. Position statement: Utility, limitations, and pitfalls in measuring testosterone: an Endocrine Society position statement. J Clin Endocrinol Metab. 2007;92(2):405–13. 10.1210/jc.2006-1864. 17090633 10.1210/jc.2006-1864

[17] Yildiz BO, Knochenhauer ES, Azziz R. Impact of obesity on the risk for polycystic ovary syndrome. J Clin Endocrinol Metab. 2008;93(1):162–8. 10.1210/jc.2007-1834. 17925334 10.1210/jc.2007-1834 PMC2190739

[18] DeUgarte CM, Woods KS, Bartolucci AA, Azziz R. Degree of facial and body terminal hair growth in unselected black and white women: toward a populational definition of hirsutism. J Clin Endocrinol Metab. 2006;91(4):1345–50. 10.1210/jc.2004-2301. 16449347 10.1210/jc.2004-2301

[19] Philpott MP, Sanders DA, Kealey T. Effects of insulin and insulin-like growth factors on cultured human hair follicles: IGF-I at physiologic concentrations is an important regulator of hair follicle growth in vitro. J Invest Dermatol. 1994;102(6):857–61. 10.1111/1523-1747.ep12382494. 8006448 10.1111/1523-1747.ep12382494

[20] Azziz R, Carmina E, Sawaya ME. Idiopathic hirsutism. Endocr Rev. 2000;21(4):347–62. 10.1210/edrv.21.4.0401. 10950156 10.1210/edrv.21.4.0401

[21] Song DK, Lee H, Hong YS, Sung Y-A. Insulin resistance is associated with hirsutism in unselected reproductive-aged women. Clin Endocrinol (Oxf). 2019;90(4):586–91. 10.1111/cen.13936. 30657205 10.1111/cen.13936

[22] Legro RS, Castracane VD, Kauffman RP. Detecting insulin resistance in polycystic ovary syndrome: purposes and pitfalls. Obstet Gynecol Surv. 2004;59(2):141. 10.1097/01.OGX.0000109523.25076.E2. 14752302 10.1097/01.OGX.0000109523.25076.E2

[23] Lizneva D, Gavrilova-Jordan L, Walker W, Azziz R. Androgen excess: Investigations and management. Best Pract Res Clin Obstet Gynaecol. 2016;37:98–118. 10.1016/j.bpobgyn.2016.05.003. 27387253 10.1016/j.bpobgyn.2016.05.003

[24] Carroll AE. The high costs of unnecessary care. JAMA. 2017;318(18):1748–9. 10.1001/jama.2017.16193. 29136432 10.1001/jama.2017.16193

[25] Gibson-Helm ME, Lucas IM, Boyle JA, Teede HJ. Women’s experiences of polycystic ovary syndrome diagnosis. Fam Pract. 2014;31(5):545–9. 10.1093/fampra/cmu028. 24925927 10.1093/fampra/cmu028

[26] Gibson-Helm M, Teede H, Dunaif A, Dokras A. Delayed diagnosis and a lack of information associated with dissatisfaction in women with polycystic ovary syndrome. J Clin Endocrinol Metab. 2017;102(2):604–12. 27906550 10.1210/jc.2016-2963 PMC6283441

[27] Ismayilova M, Yaya S. “I felt like she didn’t take me seriously”: a multi-methods study examining patient satisfaction and experiences with polycystic ovary syndrome (PCOS) in Canada. BMC Womens Health. 2022;22(1). 10.1186/s12905-022-01630-3 10.1186/s12905-022-01630-3 PMC8864824 35197027

[28] VanHise K, Wang ET, Norris K, Azziz R, Pisarska MD, Chan JL. Racial and ethnic disparities in polycystic ovary syndrome. Fertil Steril. 2023;119(3):348–54. 10.1016/j.fertnstert.2023.01.031. 36702345 10.1016/j.fertnstert.2023.01.031 PMC11354608

[29] VanHise K, et al. Regional variation in hormonal and metabolic parameters of white and black women with PCOS in the United States. J Clin Endocrinol Metab. 2023;108(3):706–12. 10.1210/clinem/dgac515. 36218376 10.1210/clinem/dgac515 PMC10210617

[30] Kushnir VA, Halevy N, Barad DH, Albertini DF, Gleicher N. Relative importance of AMH and androgens changes with aging among non-obese women with polycystic ovary syndrome. J Ovarian Res. 2015;8(1):45. 10.1186/s13048-015-0175-x. 26156856 10.1186/s13048-015-0175-x PMC4496928

[31] Xu Y, Qiao J. Association of insulin resistance and elevated androgen levels with polycystic ovarian syndrome (PCOS): a review of literature. J Healthc Eng. 2022;2022:9240569. 10.1155/2022/9240569. 35356614 10.1155/2022/9240569 PMC8959968

[32] Wild R. Long-term health consequences of PCOS. Hum Reprod UPDATE. 2002;8(3):231–41. 10.1093/humupd/8.3.231. 12078834 10.1093/humupd/8.3.231

[33] Bril F, et al. Adipose tissue dysfunction in polycystic ovary syndrome. J Clin Endocrinol Metab. 2024;109(1):10–24. 10.1210/clinem/dgad356. 10.1210/clinem/dgad356 PMC10735305 37329216

[34] Daniels K, Abma JC. Current contraceptive status among women aged 15-49: United States, 2017-2019. NCHS Data Brief No. 388. 2020 33151146

[35] Alabama Department of Public Health. Obesity trends (data). 2024. Available: https://www.alabamapublichealth.gov/awa/trends.html. Accessed Jan. 27, 2024

[36] U.S. Census Bureau. QuickFacts: Birmingham city, Alabama. Available: https://www.census.gov/quickfacts/fact/table/birminghamcityalabama/PST045223. Accessed Jan. 27, 2024

